# Discovery of Novel Cysteine Protease Inhibitors for the Treatment of Coronavirus (COVID-19)

**DOI:** 10.2174/0929867330666230519163305

**Published:** 2024-10-23

**Authors:** Surya K. De

**Affiliations:** 1 Department of Chemistry, Conju-Probe, San Diego, California, USA;; 2 Department of Chemistry, Bharath University, Chennai, Tamil Nadu, 600126, India

**Keywords:** Cysteine protease inhibitors, COVID-19, cysteine proteases, 3C-like protease, paxlovid, molnupiravir

## Abstract

The application describes compounds, such as compounds of general Formula, with warheads and their use in treating medical diseases or disorders, such as viral infections. Pharmaceutical compositions and synthetic methods of various compounds with warheads are included. The compounds are inhibitors of proteases, such as the 3C, CL- or 3CL-like protease.

## 1 COMPOUND CLASS (GENERAL FORMULA)


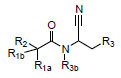


**Title:** Inhibitors of cysteine proteases and methods of use there of

**Patent Publication Number:** US11312704B2

**URL:** https://patents.google.com/patent/US11312704B2/en?oq=US11312704B2

**Patent Grant Date:** April 26, 2022

**Patent Priority Number:** US202063012039P

**Priority Date:** April 17, 2020

**Inventors:** Arnold, L. D.; Jennings, A.; Keung, W.

**Assignee Company:** Pardes Biosciences Inc, San Diego, California, USA

**Disease Area:** Coronavirus and other viruses

**Biological Target:** SARS-CoV-2 main protease and HCoV 229E

**Number of Claims:** 7

**Number of Compounds Claims:** 781

## 2 SUMMARY

The Coronaviridae family of viruses are enveloped, single-stranded, and positive-sense RNA viruses. There are 141 species that are categorized into four general groups according to their phylogenetic relationships: α-, β-, γ-, and δ-coronavirus. Coronaviruses (CoVs) are zoonotic viruses. These viruses infect a variety of animals from whales to birds, bats, cats, and humans resulting in mild to moderate respiratory tract infections [1-12]. Severe acute respiratory syndrome coronavirus (SARS-CoV) is a human coronavirus that was affected in the first pandemic of the 21^st^ century, resulting in over 8,000 people with a 10% mortality rate. Middle East respiratory syndrome coronavirus (MERS-CoV) was infected in November 2012 and has since infected over 1,600 people in 26 countries with a 36% mortality rate. Recently, COVID-19 (SARS CoV2) coronavirus became a global pandemic and it was first identified in China in 2019. As of 6 April 2023, there have been 762,201,169 confirmed cases of COVID-19, and 6,893,190 deaths globally according to the World Health Organization. It is an urgent and unmet need to discover a new broad-spectrum anti-coronaviral agent to treat the current infections and emerging coronaviruses in the future.

Most coronaviruses have >800 kDa replicase polyprotein, two or three cysteine proteases, the papain-like proteases (PLPpro, or PLP1 and PLP2), and the 3C-like protease (3CLpro, nsp5, or Mpro). These proteases make the CoV replicase polyprotein by cleaving it into 16 non-structural proteins. The CoV 3CLpro makes 11 cleavage sites within the replicase polyprotein and is essential for CoV survival. The overall active site architecture and substrate recognition pockets are structurally conserved across CoV 3CLpros, making it a valid target for the development of broad-spectrum anti-CoV therapeutics.

The sulfur of the cysteine in the protease behaves as a nucleophile and an electrophilic nitrile moiety consists of this series of compounds in the C-terminus. When the compound binds into the 3CLpro active site, the Cys145 thiol group attacks on the nitrile group, forming a covalent bond formation thus inhibiting the viral protease activities as shown in Fig. (**1**).

## 3 DEFINITIONS

From the general formula, wherein:

R_1a_ is selected from hydrogen and C_1_-C_8_alkyl;

R_1b_ is selected from hydrogen and C_1_-C_8_alkyl;

R_1a_ and R_2_ are joined together to form, together with the carbon to which they are attached, a 4-10 membered monocyclic or bicyclic heterocycle having a ring nitrogen, wherein the heterocycle may be optionally substituted on a free carbon by one, two, or three substituents;

R_3_ is selected from 4-10 membered heterocycles;

R_3b_ is selected from hydrogen or C_1_-C_8_alkyl.

## 4 SYNTHESIS

**Scheme 1:**
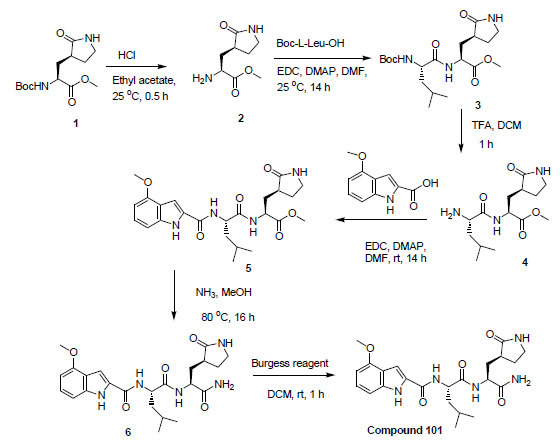
Synthesis of compound **101.**

The synthesis of compound **101** starts from compound **1** as shown in Scheme **1**. Deprotection of the Boc group in the presence of HCl in ethyl acetate provides compound **2** in 74% yield. Compound **2** is coupled with Boc-L-Leu-OH using EDC, and DMAP in DMF for 14 h to give compound **3** in 75% yield. The Boc group is removed in the presence of TFA in DCM to afford compound **4** in 84% yield. Compound **4** is coupled with 4-methoxy-1*H*-indole-2-carboxylic acid using EDC, and DMAP in DMF to give compound **5** in 48% yield. The Methyl ester of compound **5** is converted to the corresponding amide using ammonia in methanol to give compound **6** in 76% yield. The conversion of amide to the nitrile using Burgess reagent provides the final compound **101** in 25% yield.

## 5 KEY STRUCTURES

This series of compounds has three moieties as shown in Fig. (**2**). Indole is attached to a leucine *via* an amide bond followed by 2-oxopyrrolidine-3-yl nitrile with another amide bond.

## 6 KEY COMPOUNDS

The chemical structures of the most potent compounds are shown in Fig. (**3**).

## 7 BIOLOGICAL ASSAY

### 7.1 Evaluation of Antiviral Activity of Compounds against COVID-19 (nCoV-2019, SARS-CoV2) Mpro in the Enzymatic Assay

Compounds were assayed using standard methods to determine compound activity and IC_50_ values. This patent provided a detailed experimental procedure.

Evaluation of antiviral activity of compounds against human Coronavirus (HCov) 229E and OC43 in the Cytopathic Effect (CPE) assays, for detailed experimental procedures, please read the patent.

### 7.2 Biological Data

Key compounds’ inhibition data are summarized in Table **1**.

A > 30 μM; B > 10 μM and ≤ 30 μM; C ≥ 2 μM and ≤ 10 μM; D < 2 μM.

## CONCLUSION

This application provides several potent small molecules inhibiting COVID-19 and human coronavirus. Currently, Paxlovid and Molnupiravir are used to treat coronavirus infection. Both drugs have some adverse reactions and some patients get rebound effects. There are no complete cure medicines to tackle any pandemic. There is an urgent need for a potent drug with fewer adverse reactions. These small molecules have high drug-like properties with a good inhibition profile. We anticipate these compounds could be the potential benefit for the treatment of COVID-19, human coronavirus, and other viruses. The key compounds have a selectivity index (CC_50_/EC_50_) >50 times.

## Figures and Tables

**Fig. (1):**
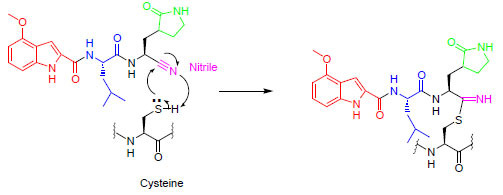
Modification of Cysteine of the protease with nitrile group of the compound, forming a covalent bond.

**Fig. (2):**
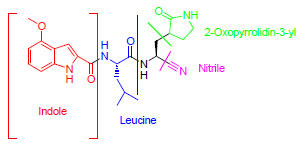
Chemical structure of compound **101**.

**Fig. (3):**
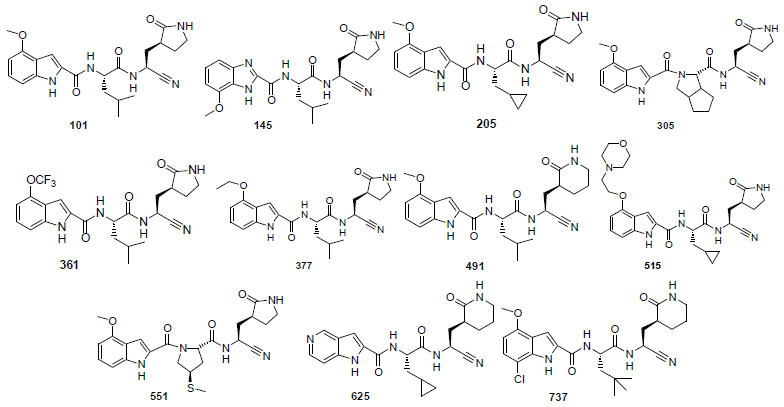
Selected potent compounds against COVID-19 and human coronavirus.

**Table 1:** Inhibition data for key compounds against COVID-19 and human coronavirus.

**Compound Number**	**229E CPE** **EC_50_ (μM)**	**HCoV 229E, Mpro** **IC_50_ (µM)**	**SARS-CoV-2, (COVID-19)** **Mpro** **IC_50_ (µM)**
101	D	D	D
145	Not tested	D	D
205	D	D	D
305	Not tested	D	D
361	D	D	D
377	D	D	D
491	D	D	D
515	Not tested	D	D
551	C	D	D
625	Not tested	D	D
737	D	D	D

## References

[1] Ábrányi-Balogh P., Petri L., Imre T., Szijj P., Scarpino A., Hrast M. (2018). Mitrović A.; Fonovič U.P.; Németh, K.; Barreteau, H.; Roper, D.I.; Horváti, K.; Ferenczy, G.G.; Kos, J.; Ilaš, J.; Gobec, S.; Keserű G.M. A road map for prioritizing warheads for cysteine targeting covalent inhibitors.. Eur. J. Med. Chem..

[2] Yu W., Zhao Y., Ye H., Wu N., Liao Y., Chen N., Li Z., Wan N., Hao H., Yan H., Xiao Y., Lai M. (2022). Structure-based design of a dual-targeted covalent inhibitor against papain-like and main proteases of SARS-CoV-2.. J. Med. Chem..

[3] Ullrich S., Nitsche C. (2022). SARS‐CoV‐2 papain‐like protease: Structure, function and inhibition.. ChemBioChem.

[4] Alzyoud L., Ghattas M.A., Atatreh N. (2022). Allosteric binding sites of the SARS-CoV-2 main protease: Potential targets for broad-spectrum anti-coronavirus agents.. Drug Des. Devel. Ther..

[5] Cherqaoui D., Oubahmane M., Hdoufane I., Bjij I., Lahcen N.A., Villemin D., Daoud R., Allali A.E. (2022). Host cell proteases mediating SARS-CoV-2 entry: An overview.. Curr. Top. Med. Chem..

[6] Hu Q., Xiong Y., Zhu G.H., Zhang Y.N., Zhang Y.W., Huang P., Ge G.B. (2020). The SARS-CoV-2 main protease (Mpro): Structure, function, and emerging therapies for COVID-19.. MedComm.

[7] Ng T.I., Correia I., Seagal J., DeGoey D.A., Schrimpf M.R., Hardee D.J., Noey E.L., Kati W.M. (2022). Antiviral drug discovery for the treatment of COVID-19 infections.. Viruses.

[8] Cannalire R., Cerchia C., Beccari A.R., Di Leva F.S., Summa V. (2022). Targeting SARS-CoV-2 proteases and polymerase for COVID-19 treatment: State of the art and future opportunities.. J. Med. Chem..

[9] Adedeji A.O., Sarafianos S.G. (2014). Antiviral drugs specific for coronaviruses in preclinical development.. Curr. Opin. Virol..

[10] Amin S.A., Banerjee S., Ghosh K., Gayen S., Jha T. (2021). Protease targeted COVID-19 drug discovery and its challenges: Insight into viral main protease (Mpro) and papain-like protease (PLpro) inhibitors.. Bioorg. Med. Chem..

[11] Dragovich P.S., Webber S.E., Babine R.E., Fuhrman S.A., Patick A.K., Matthews D.A., Lee C.A., Reich S.H., Prins T.J., Marakovits J.T., Littlefield E.S., Zhou R., Tikhe J., Ford C.E., Wallace M.B., Meador J.W., Ferre R.A., Brown E.L., Binford S.L., Harr J.E.V., DeLisle D.M., Worland S.T. (1998). Structure-based design, synthesis, and biological evaluation of irreversible human rhinovirus 3C protease inhibitors. 1. Michael acceptor structure-activity studies.. J. Med. Chem..

[12] Vandyck K., Abdelnabi R., Gupta K., Jochmans D., Jekle A., Deval J., Misner D., Bardiot D., Foo C.S., Liu C., Ren S., Beigelman L., Blatt L.M., Boland S., Vangeel L., Dejonghe S., Chaltin P., Marchand A., Serebryany V., Stoycheva A., Chanda S., Symons J.A., Raboisson P., Neyts J. (2021). ALG-097111, a potent and selective SARS-CoV-2 3-chymotrypsin-like cysteine protease inhibitor exhibits in vivo efficacy in a Syrian hamster model.. Biochem. Biophys. Res. Commun..

